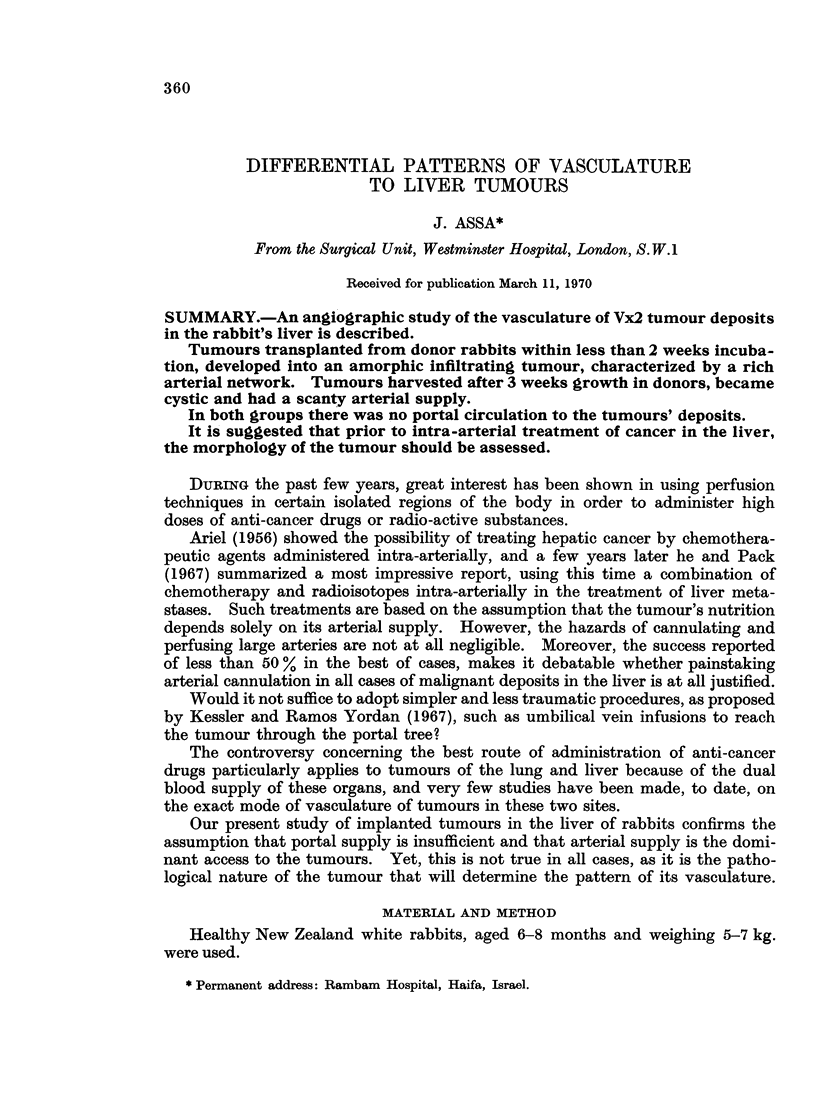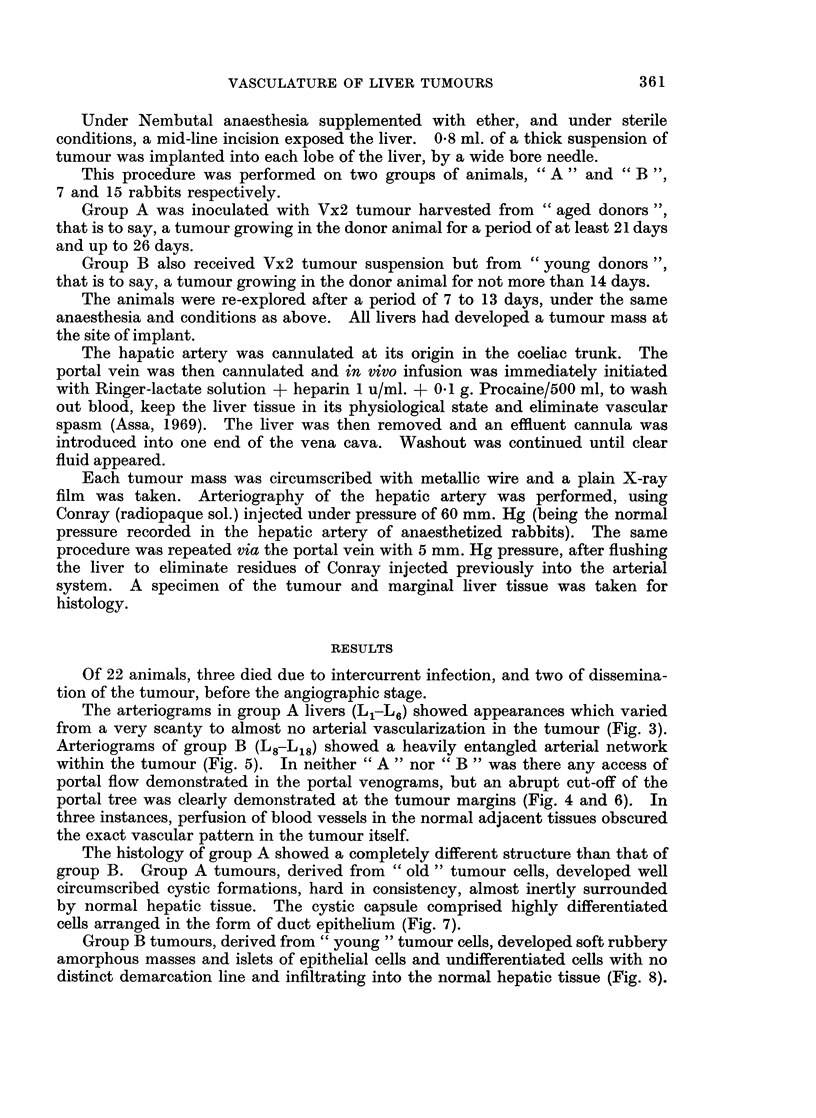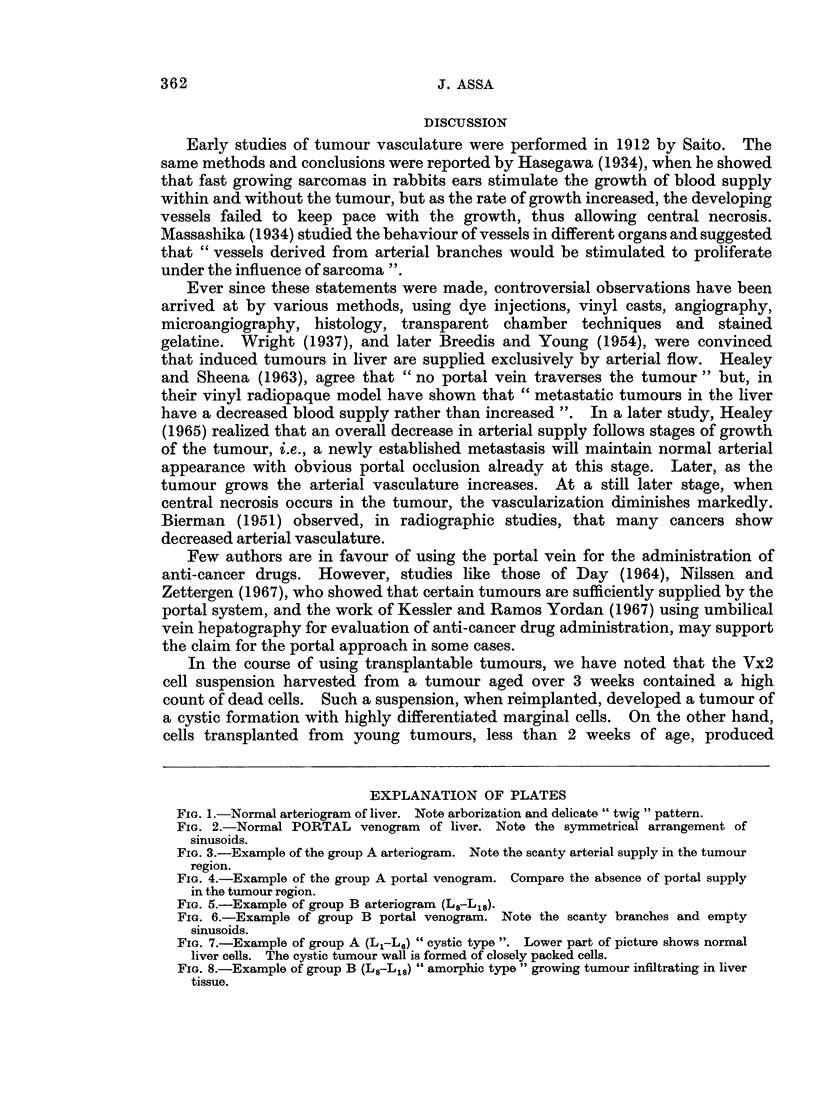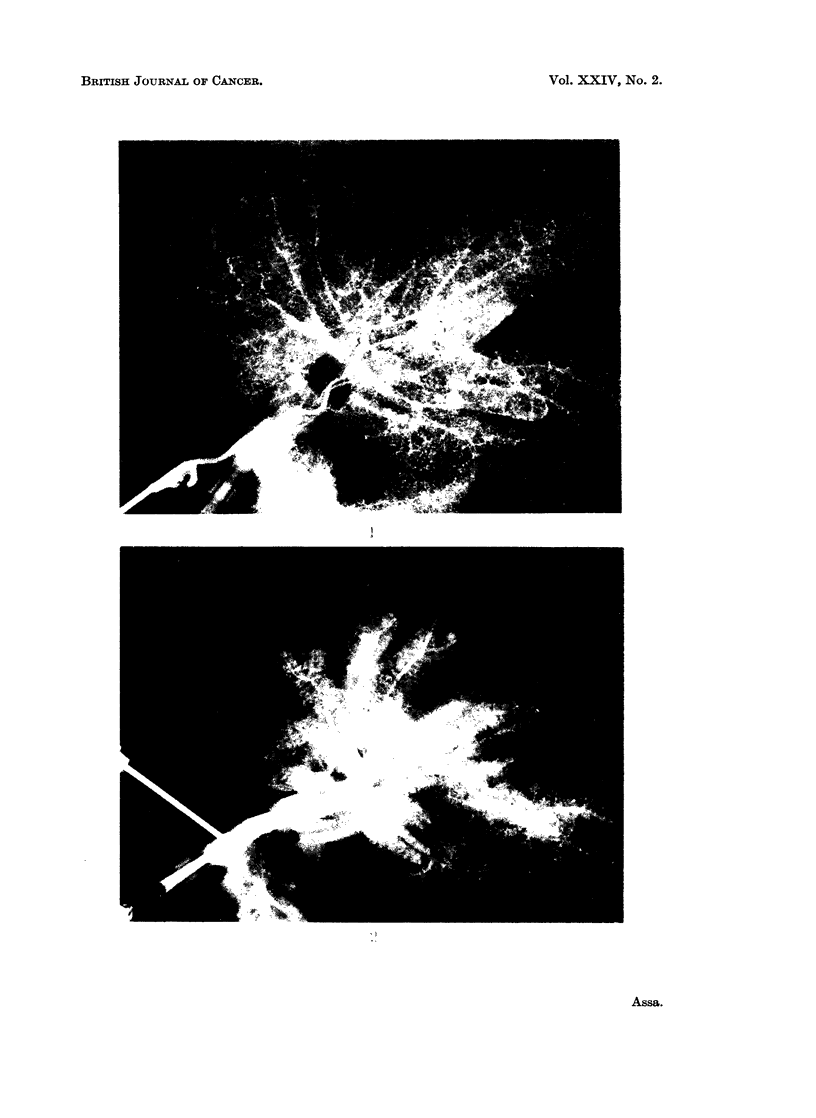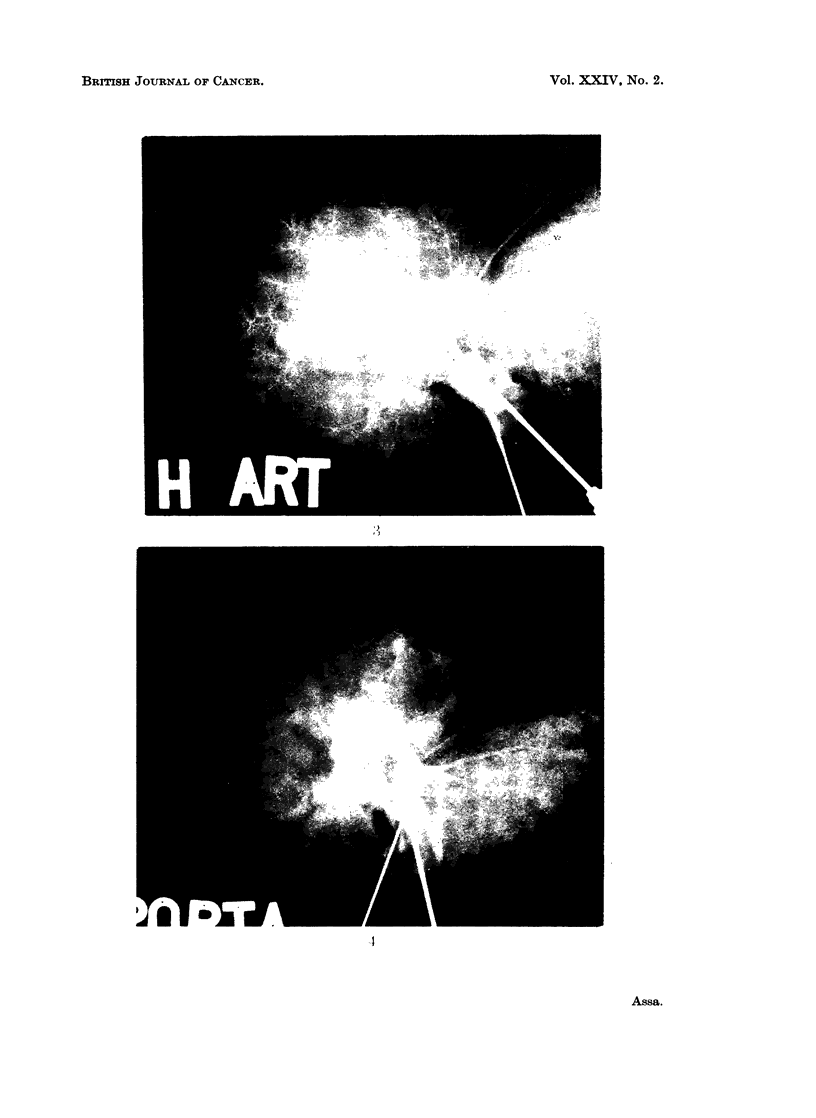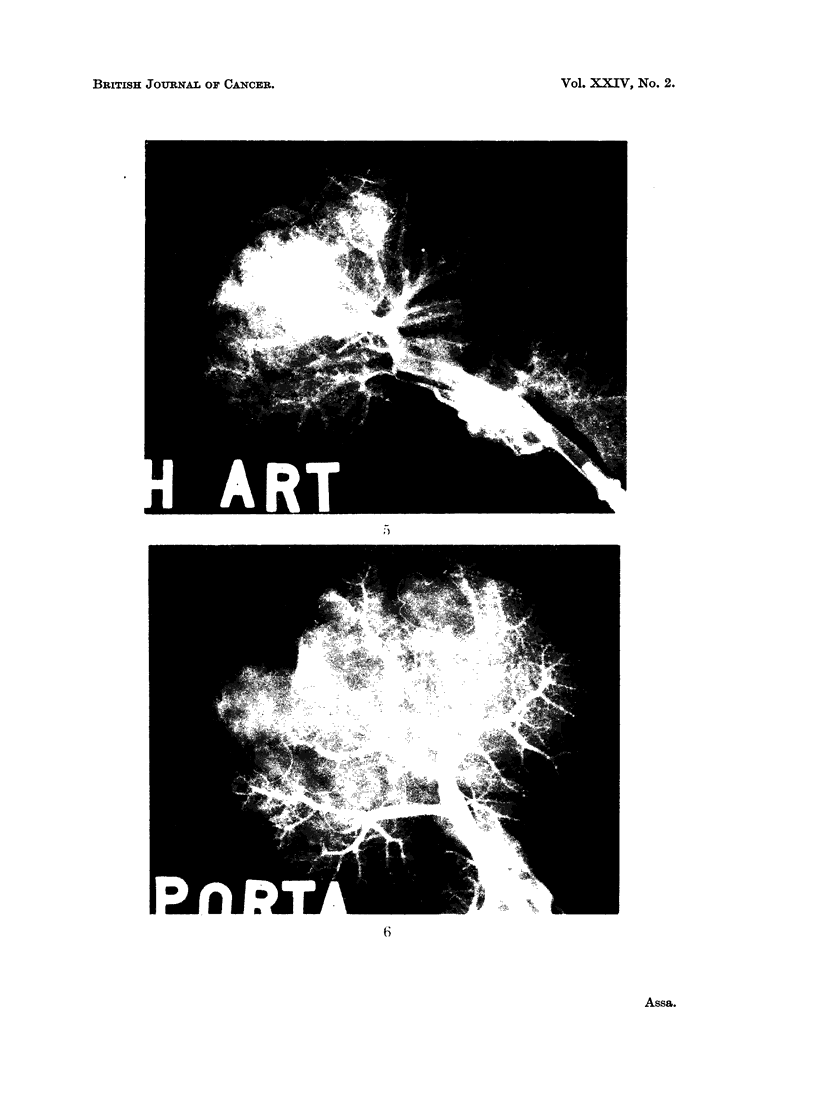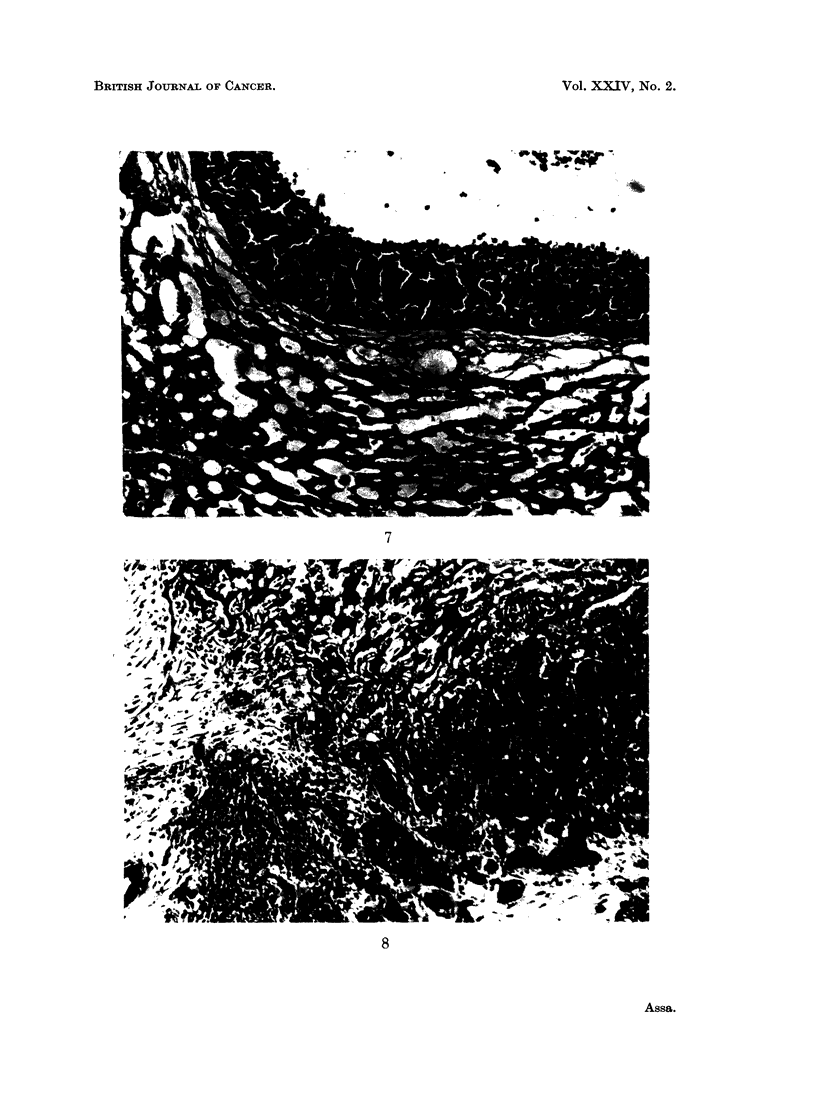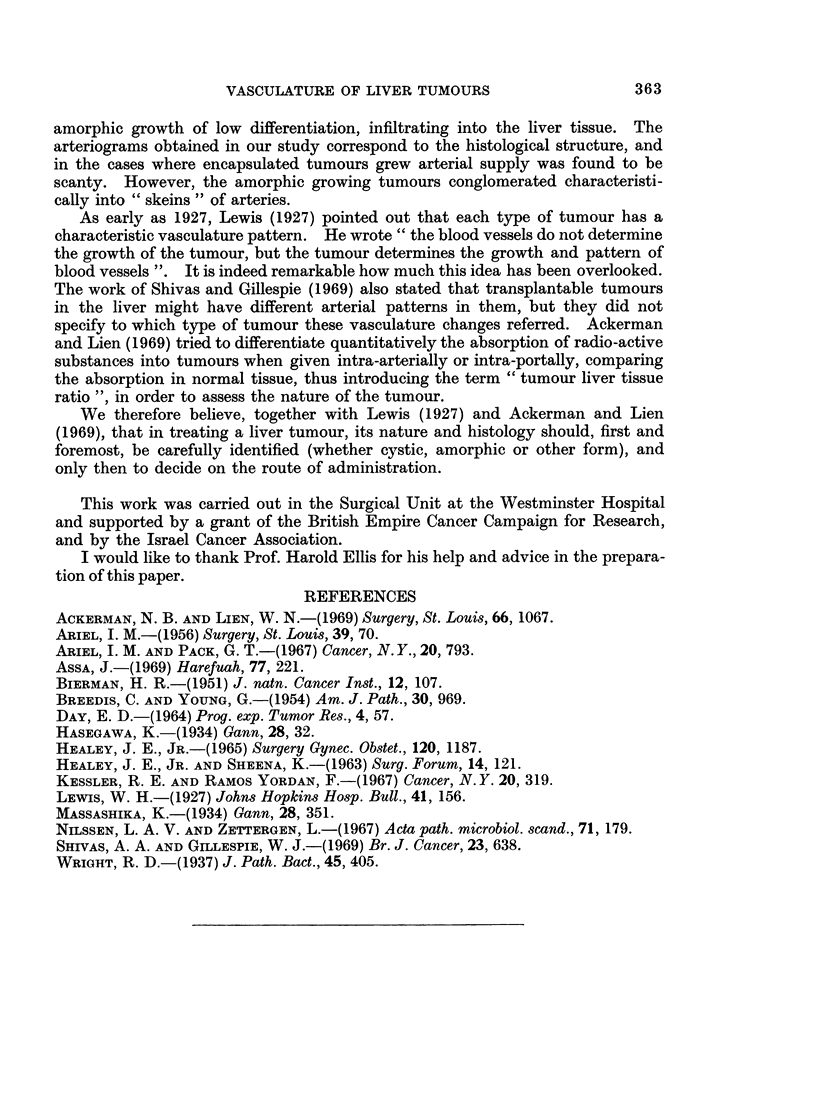# Differential Patterns of Vasculature to Liver Tumours

**DOI:** 10.1038/bjc.1970.41

**Published:** 1970-06

**Authors:** J. Assa

## Abstract

**Images:**


### DIFFERENTIAL PATTERNS OF VASCULATURE TO LIVER TUMOURS

J. ASSA*

*From the Surgical Unit, Westminster Hospital, London, S.W.1*

Received for publication March 11, 1970

**SUMMARY.—An angiographic study of the vasculature of Vx2 tumour deposits in the rabbit's liver is described.**

**Tumours transplanted from donor rabbits within less than 2 weeks incubation, developed into an amorphic infiltrating tumour, characterized by a rich arterial network. Tumours harvested after 3 weeks growth in donors, became cystic and had a scanty arterial supply.**

**In both groups there was no portal circulation to the tumours' deposits.**

**It is suggested that prior to intra-arterial treatment of cancer in the liver, the morphology of the tumour should be assessed.**

DURING the past few years, great interest has been shown in using perfusion techniques in certain isolated regions of the body in order to administer high doses of anti-cancer drugs or radio-active substances.

Ariel (1956) showed the possibility of treating hepatic cancer by chemotherapeutic agents administered intra-arterially, and a few years later he and Pack (1967) summarized a most impressive report, using this time a combination of chemotherapy and radioisotopes intra-arterially in the treatment of liver metastases. Such treatments are based on the assumption that the tumour's nutrition depends solely on its arterial supply. However, the hazards of cannulating and perfusing large arteries are not at all negligible. Moreover, the success reported of less than 50% in the best of cases, makes it debatable whether painstaking arterial cannulation in all cases of malignant deposits in the liver is at all justified.

Would it not suffice to adopt simpler and less traumatic procedures, as proposed by Kessler and Ramos Yordan (1967), such as umbilical vein infusions to reach the tumour through the portal tree?

The controversy concerning the best route of administration of anti-cancer drugs particularly applies to tumours of the lung and liver because of the dual blood supply of these organs, and very few studies have been made, to date, on the exact mode of vasculature of tumours in these two sites.

Our present study of implanted tumours in the liver of rabbits confirms the assumption that portal supply is insufficient and that arterial supply is the dominant access to the tumours. Yet, this is not true in all cases, as it is the pathological nature of the tumour that will determine the pattern of its vasculature.

### MATERIAL AND METHOD

Healthy New Zealand white rabbits, aged 6–8 months and weighing 5–7 kg. were used.

* Permanent address: Rambam Hospital, Haifa, Israel.

Under Nembutal anaesthesia supplemented with ether, and under sterile conditions, a mid-line incision exposed the liver. 0·8 ml. of a thick suspension of tumour was implanted into each lobe of the liver, by a wide bore needle.

This procedure was performed on two groups of animals, " A " and " B ", 7 and 15 rabbits respectively.

Group A was inoculated with Vx2 tumour harvested from " aged donors ", that is to say, a tumour growing in the donor animal for a period of at least 21 days and up to 26 days.

Group B also received Vx2 tumour suspension but from " young donors ", that is to say, a tumour growing in the donor animal for not more than 14 days.

The animals were re-explored after a period of 7 to 13 days, under the same anaesthesia and conditions as above. All livers had developed a tumour mass at the site of implant.

The hapatic artery was cannulated at its origin in the coeliac trunk. The portal vein was then cannulated and *in vivo* infusion was immediately initiated with Ringer-lactate solution + heparin 1 u/ml. + 0·1 g. Procaine/500 ml, to wash out blood, keep the liver tissue in its physiological state and eliminate vascular spasm (Assa, 1969). The liver was then removed and an effluent cannula was introduced into one end of the vena cava. Washout was continued until clear fluid appeared.

Each tumour mass was circumscribed with metallic wire and a plain X-ray film was taken. Arteriography of the hepatic artery was performed, using Conray (radiopaque sol.) injected under pressure of 60 mm. Hg (being the normal pressure recorded in the hepatic artery of anaesthetized rabbits). The same procedure was repeated *via* the portal vein with 5 mm. Hg pressure, after flushing the liver to eliminate residues of Conray injected previously into the arterial system. A specimen of the tumour and marginal liver tissue was taken for histology.

### RESULTS

Of 22 animals, three died due to intercurrent infection, and two of dissemination of the tumour, before the angiographic stage.

The arteriograms in group A livers ($L_1$–$L_6$) showed appearances which varied from a very scanty to almost no arterial vascularization in the tumour (Fig. 3). Arteriograms of group B ($L_8$–$L_{18}$) showed a heavily entangled arterial network within the tumour (Fig. 5). In neither " A " nor " B " was there any access of portal flow demonstrated in the portal venograms, but an abrupt cut-off of the portal tree was clearly demonstrated at the tumour margins (Fig. 4 and 6). In three instances, perfusion of blood vessels in the normal adjacent tissues obscured the exact vascular pattern in the tumour itself.

The histology of group A showed a completely different structure than that of group B. Group A tumours, derived from " old " tumour cells, developed well circumscribed cystic formations, hard in consistency, almost inertly surrounded by normal hepatic tissue. The cystic capsule comprised highly differentiated cells arranged in the form of duct epithelium (Fig. 7).

Group B tumours, derived from " young " tumour cells, developed soft rubbery amorphous masses and islets of epithelial cells and undifferentiated cells with no distinct demarcation line and infiltrating into the normal hepatic tissue (Fig. 8).

### DISCUSSION

Early studies of tumour vasculature were performed in 1912 by Saito. The same methods and conclusions were reported by Hasegawa (1934), when he showed that fast growing sarcomas in rabbits ears stimulate the growth of blood supply within and without the tumour, but as the rate of growth increased, the developing vessels failed to keep pace with the growth, thus allowing central necrosis. Massashika (1934) studied the behaviour of vessels in different organs and suggested that " vessels derived from arterial branches would be stimulated to proliferate under the influence of sarcoma ".

Ever since these statements were made, controversial observations have been arrived at by various methods, using dye injections, vinyl casts, angiography, microangiography, histology, transparent chamber techniques and stained gelatine. Wright (1937), and later Breedis and Young (1954), were convinced that induced tumours in liver are supplied exclusively by arterial flow. Healey and Sheena (1963), agree that " no portal vein traverses the tumour " but, in their vinyl radiopaque model have shown that " metastatic tumours in the liver have a decreased blood supply rather than increased ". In a later study, Healey (1965) realized that an overall decrease in arterial supply follows stages of growth of the tumour, *i.e.*, a newly established metastasis will maintain normal arterial appearance with obvious portal occlusion already at this stage. Later, as the tumour grows the arterial vasculature increases. At a still later stage, when central necrosis occurs in the tumour, the vascularization diminishes markedly. Bierman (1951) observed, in radiographic studies, that many cancers show decreased arterial vasculature.

Few authors are in favour of using the portal vein for the administration of anti-cancer drugs. However, studies like those of Day (1964), Nilssen and Zettergen (1967), who showed that certain tumours are sufficiently supplied by the portal system, and the work of Kessler and Ramos Yordan (1967) using umbilical vein hepatography for evaluation of anti-cancer drug administration, may support the claim for the portal approach in some cases.

In the course of using transplantable tumours, we have noted that the Vx2 cell suspension harvested from a tumour aged over 3 weeks contained a high count of dead cells. Such a suspension, when reimplanted, developed a tumour of a cystic formation with highly differentiated marginal cells. On the other hand, cells transplanted from young tumours, less than 2 weeks of age, produced

---

### EXPLANATION OF PLATES

FIG. 1.—Normal arteriogram of liver. Note arborization and delicate " twig " pattern.

FIG. 2.—Normal PORTAL venogram of liver. Note the symmetrical arrangement of sinusoids.

FIG. 3.—Example of the group A arteriogram. Note the scanty arterial supply in the tumour region.

FIG. 4.—Example of the group A portal venogram. Compare the absence of portal supply in the tumour region.

FIG. 5.—Example of group B arteriogram ($L_8$–$L_{18}$).

FIG. 6.—Example of group B portal venogram. Note the scanty branches and empty sinusoids.

FIG. 7.—Example of group A ($L_1$–$L_6$) " cystic type ". Lower part of picture shows normal liver cells. The cystic tumour wall is formed of closely packed cells.

FIG. 8.—Example of group B ($L_8$–$L_{18}$) " amorphic type " growing tumour infiltrating in liver tissue.

1

2

Assa.

3

4

Assa.

5

6

Assa.

7

8

Assa.

amorphic growth of low differentiation, infiltrating into the liver tissue. The arteriograms obtained in our study correspond to the histological structure, and in the cases where encapsulated tumours grew arterial supply was found to be scanty. However, the amorphic growing tumours conglomerated characteristically into " skeins " of arteries.

As early as 1927, Lewis (1927) pointed out that each type of tumour has a characteristic vasculature pattern. He wrote " the blood vessels do not determine the growth of the tumour, but the tumour determines the growth and pattern of blood vessels ". It is indeed remarkable how much this idea has been overlooked. The work of Shivas and Gillespie (1969) also stated that transplantable tumours in the liver might have different arterial patterns in them, but they did not specify to which type of tumour these vasculature changes referred. Ackerman and Lien (1969) tried to differentiate quantitatively the absorption of radio-active substances into tumours when given intra-arterially or intra-portally, comparing the absorption in normal tissue, thus introducing the term " tumour liver tissue ratio ", in order to assess the nature of the tumour.

We therefore believe, together with Lewis (1927) and Ackerman and Lien (1969), that in treating a liver tumour, its nature and histology should, first and foremost, be carefully identified (whether cystic, amorphic or other form), and only then to decide on the route of administration.

This work was carried out in the Surgical Unit at the Westminster Hospital and supported by a grant of the British Empire Cancer Campaign for Research, and by the Israel Cancer Association.

I would like to thank Prof. Harold Ellis for his help and advice in the preparation of this paper.

### REFERENCES

Ackerman, N. B. and Lien, W. N.—(1969) *Surgery, St. Louis*, **66**, 1067.
Ariel, I. M.—(1956) *Surgery, St. Louis*, **39**, 70.
Ariel, I. M. and Pack, G. T.—(1967) *Cancer, N.Y.*, **20**, 793.
Assa, J.—(1969) *Harefuah*, **77**, 221.
Bierman, H. R.—(1951) *J. natn. Cancer Inst.*, **12**, 107.
Breedis, C. and Young, G.—(1954) *Am. J. Path.*, **30**, 969.
Day, E. D.—(1964) *Prog. exp. Tumor Res.*, **4**, 57.
Hasegawa, K.—(1934) *Gann*, **28**, 32.
Healey, J. E., Jr.—(1965) *Surgery Gynec. Obstet.*, **120**, 1187.
Healey, J. E., Jr. and Sheena, K.—(1963) *Surg. Forum*, **14**, 121.
Kessler, R. E. and Ramos Yordan, F.—(1967) *Cancer, N.Y.* **20**, 319.
Lewis, W. H.—(1927) *Johns Hopkins Hosp. Bull.*, **41**, 156.
Massashika, K.—(1934) *Gann*, **28**, 351.
Nilssen, L. A. V. and Zettergen, L.—(1967) *Acta path. microbiol. scand.*, **71**, 179.
Shivas, A. A. and Gillespie, W. J.—(1969) *Br. J. Cancer*, **23**, 638.
Wright, R. D.—(1937) *J. Path. Bact.*, **45**, 405.